# Attenuated EAN in TNF-α Deficient Mice Is Associated with an Altered Balance of M1/M2 Macrophages

**DOI:** 10.1371/journal.pone.0038157

**Published:** 2012-05-30

**Authors:** Hong-Liang Zhang, Mohammed Y. Hassan, Xiang-Yu Zheng, Sheikh Azimullah, Hernan Concha Quezada, Naheed Amir, Mohamed Elwasila, Eilhard Mix, Abdu Adem, Jie Zhu

**Affiliations:** 1 Department of Neurobiology, Care Sciences and Society, Karolinska Institute, Stockholm, Sweden; 2 Department of Pharmacology, Faculty of Medicine and Health Sciences, United Arab Emirates University, Al Ain, United Arab Emirates; 3 Center for Infectious Medicine, Department of Medicine, Karolinska Institute, Karolinska University Hospital Huddinge, Stockholm, Sweden; 4 Department of Neurology, University of Rostock, Rostock, Germany; 5 Department of Neurology, First Hospital of Jilin University, Changchun, China; Charité-Universitätsmedizin Berlin, Germany

## Abstract

The role of tumor necrosis factor (TNF)-α and its receptors in neuroautoimmune and neuroinflammatory diseases has been controversial. On the basis of our previous studies, we hereby aimed to further clarify TNF-α’s mechanism of action and to explore the potential role of TNF-α receptor (TNFR)1 as a therapeutic target in experimental autoimmune neuritis (EAN). EAN was induced by immunization with P0 peptide 180–199 in TNF-α knockout (KO) mice and anti-TNFR1 antibodies were used to treat EAN. Particularly, the effects of TNF-α deficiency and TNFR1 blockade on macrophage functions were investigated. The onset of EAN in TNF-α KO mice was markedly later than that in wild type (WT) mice. From day 14 post immunization, the clinical signs of TNF-α KO mice were significantly milder than those of their WT counterparts. Further, we showed that the clinical severity of WT mice treated with anti-TNFR1 antibodies was less severe than that of the control WT mice receiving PBS. Nevertheless, no difference with regard to the clinical signs of EAN or inflammatory infiltration in cauda equina was seen between TNF-α KO and WT mice with EAN after blockade of TNFR1. Although TNF-α deficiency did not alter the proliferation of lymphocytes in response to either antigenic or mitogenic stimuli, it down-regulated the production of interleukin (IL)-12 and nitric oxide (NO), and enhanced the production of IL-10 in macrophages. Increased ratio of regulatory T cells (Tregs) and reduced production of interferon (IFN)-γ in cauda equina infiltrating cells, and elevated levels of IgG2b antibodies against P0 peptide 180–199 in sera were found in TNF-α KO mice with EAN. In conclusion, TNF-α deficiency attenuates EAN via altering the M1/M2 balance of macrophages.

## Introduction

Guillain-Barré syndrome (GBS) is an inflammatory demyelinating disorder of the peripheral nervous system (PNS) in humans. The clinical feature of GBS is characterized by rapidly progressive weakness and sensory dysfunction in the limbs as well as respiratory weakness [1]. The exact pathogenesis of GBS remains largely unknown. Experimental autoimmune neuritis (EAN) shares clinical, histopathological, and electrophysiological features with GBS and hence can be employed as an animal model to explore its pathogenesis. Pathologically, EAN is attributable to breakdown of the blood-nerve barrier (BNB), robust accumulation of reactive T cells and macrophages in the PNS and demyelination of peripheral nerves [2]. In order to cross the BNB and to provoke a local inflammatory response, circulating autoreactive T cells need to be activated and to produce proinflammatory cytokines, including tumor necrosis factor (TNF)-α, interleukin (IL)-1 and IL-6 [3]. Macrophages are the predominant cell population in the lesions of GBS and EAN [4]. As professional antigen presenting cells, macrophages express major histocompatibility complex (MHC)-II and co-stimulatory B7 molecules, and thus are critical in the activation of T helper (Th) cells and the triggering of the autoimmune process. Moreover, macrophages are crucial in the effector phase of EAN via phagocytotic attack and secretion of inflammatory mediators such as TNF-α and nitric oxide (NO) [5].

TNF-α is a Th1 cytokine that is expressed on activated macrophages, T cells, NK cells, and, to a lesser extent, on tissue cells such as endothelial cells, smooth muscle cells, fibroblasts, astrocytes, neurons and Schwann cells (SCs). Depending on binding to different TNF-α receptors (TNFRs), i.e. TNFR1 or TNFR2, TNF-α bears proinflammatory or antiinflammatory properties [6]. Polymorphisms of TNF-α and its promoter have been associated with susceptibility to GBS [7]. TNF-α has been identified as a key player in the pathogenesis of GBS and EAN. Clinically, an increased level of TNF-α in serum has been correlated with the disease severity of GBS [8], [9]. Likewise, levels of TNF-α in serum decrease after immunomodulatory treatment and are in parallel with clinical recovery of GBS patients [10]. Expression of TNF-α mRNA in the PNS is upregulated at nadir of clinical EAN [11]. TNF-α positive macrophages appear in peripheral nerves around the onset of EAN [12]. Levels of TNF-α producing cells in blood, lymph nodes and PNS tissue roughly parallel the clinical severity of EAN. Moreover, injection of TNF-α into rat sciatic nerves resulted in inflammatory vascular changes within the endoneurium along with demyelination and axonal degeneration [13]. Furthermore, systemic administration of TNF-α markedly worsened EAN [14]. Conversely, treatment with monoclonal antibodies (mAb) against TNF-α or soluble TNFR1 ameliorated EAN [15], [16]. In addition, antiinflammatory compounds such as rolipram, linomede and leflunomide markedly inhibited cellular infiltration and downregulated production of TNF-α, and by doing so, suppressed the clinical symptoms of EAN [17]–[19].

To further clarify the role of TNF-α in the pathogenesis of EAN and to explore the potential of TNFR1 as a therapeutic target in GBS, we induced EAN in TNF-α KO mice and studied the effect of TNFR1 blockade with anti-TNFR1 antibodies on EAN. Specifically, the effects of TNF-α deficiency and TNFR1 blockade on macrophage functions were investigated.

## Materials and Methods

### Ethics Statement

The EAN model on mice was approved by the South Stockholm Research Animal Ethics Committee, Huddinge County Court, Stockholm (S40-11) and the Animal Research Ethics Committee, Faculty of Medicine & Health Sciences, United Arab Emirates (UAE) University, Al Ain, UAE (A11/11).

### Animals

TNF-α KO and and wild type (WT) mice were purchased from Taconic (Taconic, Ry, Demark) and housed at the animal facility of the Faculty of Medicine and Health Sciences, UAE University, Al Ain, UAE. Male mice, 5–6 weeks old were used for the study. All mice were housed on a 12/12 light-dark schedule with water and food available *ad libitum*.

### Antigen

The neuritogenic P0 peptide 180–199 (SSKRGRQTPVLYAMLDHSRS) of murine PNS myelin P0 protein was synthesized by the 9-fluorenylmethoxycarbonyl (Fmoc) solid-phase procedure [20], purified by high-performance liquid chromatography (HPLC) using a Vydac reverse-phase column (Grace Vydac, Hesperia, CA, USA), and analyzed by matrix-assisted laser desorption/ionization (MALDI) time-of-flight (TOF) mass spectrometry (Cambridge Research Biochemicals, Billingham, UK).

### Induction of the EAN Model and Assessment of the Clinical Course

Emulsion of P0 peptide and Freund’s complete adjuvant (FCA) was prepared by gently adding P0 peptide solution in 0.9% saline to FCA being vortexed. FCA refers to *Mycobacterium tuberculosis* plus Freund’s incomplete adjuvant (FIA). EAN was induced by immunizing mice twice (days 0 and 8) via subcutaneous injection of 150 µg P0 peptide 180–199, 0.5 mg *Mycobacterium tuberculosis* (strain H37 RA; Difco, Franklin Lakes, NJ, USA) in 25 µl saline, and 25 µl FIA (ICN Biomedicals, Costa Mesa, CA, USA) to the back of mice. All mice received 400, 300, and 300 ng pertussis toxin (PTX, Merck, Whitehouse Station, NJ, USA) by intravenous injection (via tail veins) on days −1, +1 and +3 post immunization (p.i.), respectively. Using a blinded protocol, two examiners assessed clinical signs of mice with EAN immediately before immunization (day 0) and thereafter every two days until day 59 p.i. EAN was scored as follows: 0, normal; 1, less lively, reduced tone of the tail; 2, flaccid tail; 3, abnormal gait; 4, ataxia; 5, mild paraparesis; 6, moderate paraparesis; 7, severe paraparesis; 0.5, intermediate clinical signs.

### Lymphocyte Proliferation Test

Mice were sacrificed at the height of EAN (day 28 p.i.) after perfusion with phosphate-buffered saline (PBS). Spleens were removed and single cell suspensions of mononuclear cells (MNCs) in RPMI-1640 (Invitrogen, Carlsbad, CA, USA) were prepared and cultured. Concanavalin A (ConA, 10 µg/ml, Sigma-Aldrich, St. Louis, MO, USA), P0 peptide 180–199 (10 µg/ml) and IL-23 (100 ng/ml, eBioscience, San Diego, CA, USA) were used as stimuli. The concentrations had maximum stimulatory effects as assessed in pilot experiments. After 60 h of cultivation with respective stimulus, the proliferation was assessed using the CellTiter 96® AQ_ueous_ One Solution Cell Proliferation Assay (Promega Corporation, Madison, WI, USA) according to the manufacturer’s instructions. Briefly, assays were performed by adding a small amount of the reagent directly to culture wells. The CellTiter 96® AQ_ueous_ One Solution Cell Proliferation Assay contains a novel tetrazolium compound [3-(4,5-dimethylthiazol-2-yl)-5-(3-carboxymethoxyphenyl)-2-(4-sulfophenyl)-2H-tetrazolium, inner salt; MTS(a)]. The MTS tetrazolium compound is bioreduced into a colored formazan product soluble in cell culture medium by NADPH or NADH produced by dehydrogenase in metabolically active cells. After 4 h incubation, the absorbance at 490 nm was recorded with an ELISA reader (Tecan, Männedorf, Switzerland).

### Isolation of Infiltrating Cells in the Cauda Equina (CE)

CE infiltrating cells were isolated according to method described by Duan, *et al* [21]. Briefly, CE fragments were carefully removed from PBS perfused mice, transferred to RPMI-1640, ground and passed through a 70 µm cell nylon mesh (Becton Dickinson, Franklin Lakes, NJ, USA). The collected cells were suspended in 27% Percoll (Amersham Biosciences AB, Uppsala, Sweden) in PBS and centrifuged at 1000×g for 30 min at 4°C. The pellets were harvested.

### Macrophage Cultures and NO Detection

Mice were sacrificed and standard lavage of the peritoneal exudates with 10 ml serum-free culture medium DMEM/F12 (Invitrogen) was performed. The lavage fluid contained peritoneal exudate mononuclear cells (PEMs), which represented mainly macrophages as tested by labeling with the macrophage marker macrosialin (mouse equivalent of human CD68). The single cell suspension was centrifuged at 300×g for 10 min. The pellets were re-suspended with DMEM/F12 supplemented with 5% fetal bovine serum (FBS, Sigma-Aldrich), 100 IU/ml penicillin and 100 µg/ml streptomycin (both from Gibco-Invitrogen, Grand Island, NY, USA). Macrophages were seeded into 5.3 cm Petri-dishes (Becton Dickinson, San Jose, CA, USA) at a concentration of 2×10^6^/ml. The cultured cells were stimulated with lipopolysaccharide (LPS, Sigma-Aldrich, 100 ng/ml), recombinant mouse interferon (IFN)-γ (Hycult Biotechnology, Uden, The Netherlands, 100 IU/ml), IL-12 (Sigma-Aldrich, 10 ng/ml), TNF-α (Sigma-Aldrich, 5 ng/ml), polyinosinic-polycytidylic acid (poly I:C, Sigma-Aldrich, 50 µg/ml), combinations thereof, LPS (500 ng/ml), and IFN-γ (500 IU/ml). After 24 h incubation, the supernatants were collected and snap-frozen for cytokine and NO detection. After removal of the supernatants, macrophages stimulated with 100 ng/ml LPS plus 100 IU/ml IFN-γ were incubated with Brefeldin A (3 µg/ml, eBioscience) in order to inhibit further cytokine secretion. After 6 h, these macrophages were harvested for subsequent flow cytometric analysis of intracellular cytokine expression. NO production was measured by detecting the supernatant levels of nitrite, the stable biological oxidation product of NO using the modified Griess reagent (Sigma-Aldrich). The detecting procedure was performed according to the manufacturer’s instructions. The concentrations of nitrite were quantified by extrapolation from the standard curve obtained by using sodium nitrite (Sigma-Aldrich) solutions at concentrations of 9, 3, 1, 0.33, 0.11, 0.033, and 0 μg/ml.

### Flow Cytometry

FITC-, PE-, APC- and PerCP-conjugated antibodies were purchased from commercial suppliers. Specifically, anti-mouse-macrosialin monoclonal antibodies were from AbD Serotec (Kidlington, UK); anti-mouse-CD3, CD4, CD8, CD25, CD16/32, CD45, NK1.1, dendritic cell marker, T cell receptor (TCR), B cell receptor (BCR), IL-4, IL-10, IL-12, IL-17A, IL-17F, IFN-γ, TNF-α, MCP-1, CXCL9, αβTCR and γδTCR monoclonal antibodies, and mouse-FoxP3 staining set were from eBioscience; and anti-mouse-CD45 monoclonal antibodies were from BD Biosciences (San Jose, CA, USA). Monoclonal antibodies against MHC-II, CD86 and TNFR2 were from Abcam (Cambridge, UK). Cells (CE infiltrating cells, splenic MNCs or PEMs) were harvested and washed with 1% bovine serum albumin (BSA, Sigma-Aldrich) in PBS. For staining of molecules with extracellular expression, cells were incubated with FITC-, PE-, APC, and/or PerCP-conjugated antibodies for 15 min at room temperature (RT). The staining of FoxP3 was according to instructions provided by the manufacturer. For staining of other molecules with intracellular expression, cells were fixed with 2% paraformaldehyde (Merck) for 15 min at RT and permeabilized with 0.5% freshly prepared saponin (Sigma-Aldrich) in PBS containing 1% BSA. The permeabilized cells were incubated with FITC-, PE-, APC, and/or PerCP-conjugated antibodies for 15 min at RT. FITC-, PE-, APC- and PerCP-conjugated isotype antibodies (from BD Biosciences) were used as negative controls. Cells were washed twice, resuspended in 1% paraformaldehyde in PBS and stored at 4°C until flow cytometric analysis with an FACSCanto™ II cytometer (Becton Dickinson) using the FACSDiva software (Becton Dickinson). Surface and intracellular molecule expressions were assessed by determining the positive cell percentage. In each experiment, cells from all groups were collected and analyzed at each time-point on the same day with the same cytometer settings. Flow cytometric data were analyzed with the CellQuest Pro software (Becton Dickinson) or FlowJo (TreeStar, Ashland, OR, USA).

### ELISA for Measuring Cytokines in Sera and Cell Culture Supernatants

A standardized procedure for the sandwich ELISA was established after optimization of experimental parameters. Coating and detecting antibodies against mouse IL-6, IL-10, IL-12, IL-17A, and IFN-γ, and recombinant mouse IL-6, IL-10, IL-12, IL-17A, and IFN-γ proteins as standards, respectively, were purchased from eBioscience. Briefly, monoclonal antibodies were coated onto standard ELISA plates (Nalge Nunc, Roskilde, Denmark) in a volume of 100 μl/well overnight at 4°C. After three washings with PBS, uncoated sites were blocked with 100 µl 10% FBS in PBS for 1 h at RT. Duplicates of samples (sera or macrophage supernatants) or of recombinant standards were added and the plates were incubated for 1 h at RT. After washing, the plates were incubated with biotinylated detecting antibody against IL-6, IL-10, IL-12, IL-17A, and IFN-γ (eBioscience), respectively, for 2 h at RT. Then avidin-conjugated horseradish peroxidase (HRP, eBioscience) was added for 30 min. Color reaction was performed with 100 µl of tetramethylbenzidine (TMB, Sigma-Aldrich) for 30 min and the reaction was terminated by adding 2 M sulfuric acid (Sigma-Aldrich). The plates were immediately read at 450 nm with an ELISA reader (Tecan). The concentrations of proteins were quantified by extrapolation from the standard curve.

### ELISA for Measurement of anti-P0 Peptide 180–199 Antibodies in Sera

Serum samples were obtained from mice at the peak of EAN disease (day 28 p.i.). Samples of groups of 8–10 mice were pooled. P0 peptide 180–199 was coated overnight at 4°C by adding 10 μg/ml in 100 μl per well onto ELISA plates. After three washings with PBS, uncoated sites were blocked with 100 µl 10% FBS in PBS for 2 h at RT. After three washings, serum samples were diluted at 1∶100 with 1% BSA in PBS, applied to plate wells and incubated for 1.5 h at RT. Then plates were incubated for 1 h with peroxidase-conjugated affinipure rabbit anti-mouse IgM, IgG, IgG1, IgG2a, IgG2b (Jackson ImmunoResearch Laboratories, West Grove, PA, USA) after dilution at 1∶5000 with 1% BSA in PBS. After three washings, the peroxidase substrate TMB (Sigma-Aldrich) was added and 15–30 min later the reaction was terminated by adding 2 M sulfuric acid (Sigma-Aldrich). The plates were read at 450 nm using an ELISA reader (Tecan).

### TNFR1 Blocking

Anti-TNFR1 monoclonal antibodies (Santa Cruz Biotechnology, Santa Cruz, CA, USA) were administered intravenously to WT mice (n = 5) with EAN at a dose of 200 µg/(kg body weight) on days −1, +3 and +7 p.i., respectively. Another group of age- and sex-matched WT mice (n = 5) received 0.9% saline as controls. Using a blinded protocol, two examiners assessed clinical signs of EAN mice every two days until day 35 p.i. according to the scale described above.

In a separate experiment, the TNFR1 blocking was performed in both TNF-α KO and WT mice after induction of EAN. The procedure with immunization and anti-TNFR1 monoclonal antibody treatment was as described above. The clinical severity of EAN was assessed until day 35 p.i. (n = 5 in both groups) and flow cytometry was used to assess the CE infiltrating cells on the day 28 p.i. in TNF-α KO and WT mice with EAN after blocking TNFR1 functions (n = 5 in both groups).

### Statistics

Data have been expressed as mean value ± standard deviation (SD). The Statistical Package for the Social Sciences 14.0 (SPSS, IBM, West Grove, PA, USA) was used for analyzing all data. One-way analysis of variance (ANOVA) and Kruskal-Wallis test were used to compare values among groups followed by Student’s t-test or Mann-Whitney u-test to compare values between groups. All tests were two-tailed, with the level of significance set to *p*<0.05.

## Results

### TNF-α Deficiency Attenuates the Clinical Severity of EAN

**Figure 1:**
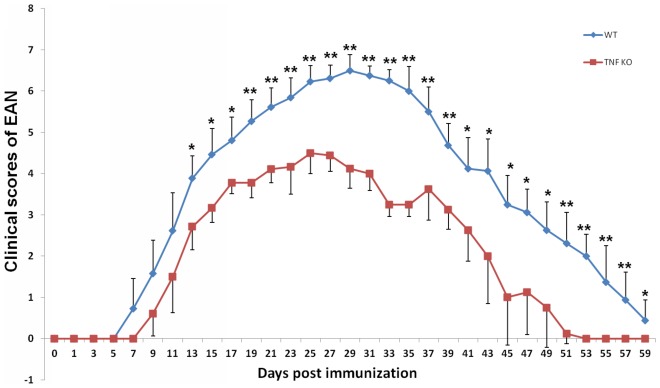
Attenuated clinical severity of EAN in TNF-α KO versus WT mice. EAN was induced in TNF-α KO mice and WT mice by immunization with P0 peptide 180–199 in combination with FCA. Using a blinded protocol, two examiners evaluated the clinical severity of EAN mice immediately before immunization (day 0 p.i.) and thereafter every two days until day 59 p.i. From day 14 p.i., the clinical signs of TNF-α KO mice were significantly milder than those of the WT controls (Mann-Whitney u-test). Data are presented as mean value ± SD (n = 12 in both groups). * *p*<0.05, ** *p*<0.01.

All mice immunized with P0 peptide 180–199 in combination with FCA acquired EAN. The onset of EAN in the TNF-α KO group was later than in WT group (9–13 days *versus* 7–9 days p.i.). EAN reached the peak of the disease on day 28 p.i. in TNF-α KO mice and on day 25 p.i. in WT mice, respectively. Disease persisted until around day 38 p.i. when evidence of recovery became discernible. Almost all the mice had recovered completely by day 59 p.i. From day 14 p.i., the clinical signs of TNF-α KO mice were significantly milder than those of their WT counterparts (Mann-Whitney u-test, all *p*<0.05, Figure 1).

### TNF-α Deficiency Does not Alter Priming Responses of Splenic MNCs

At nadir of EAN (day 28 p.i.), splenic MNCs of mice were cultured and stimulated with P0 peptide 180–199 and ConA for antigenic and mitogenic proliferation. For both sets of stimulation, the proliferation was higher in EAN mice than in naïve mice (*p*<0.05 for all comparisons, data not shown). However, there was neither any significant difference between naïve TNF-α KO and naïve WT mice, nor between EAN TNF-α KO and EAN WT mice (data not shown). There was also no significant difference between TNF-α KO and WT mice with EAN, even after stimulation with IL-23 to induce the proliferation of armed effector T cells (data not shown).

### TNF-α Deficiency Down-regulated the Proinflammatory Phenotype of Macrophages

**Figure 2:**
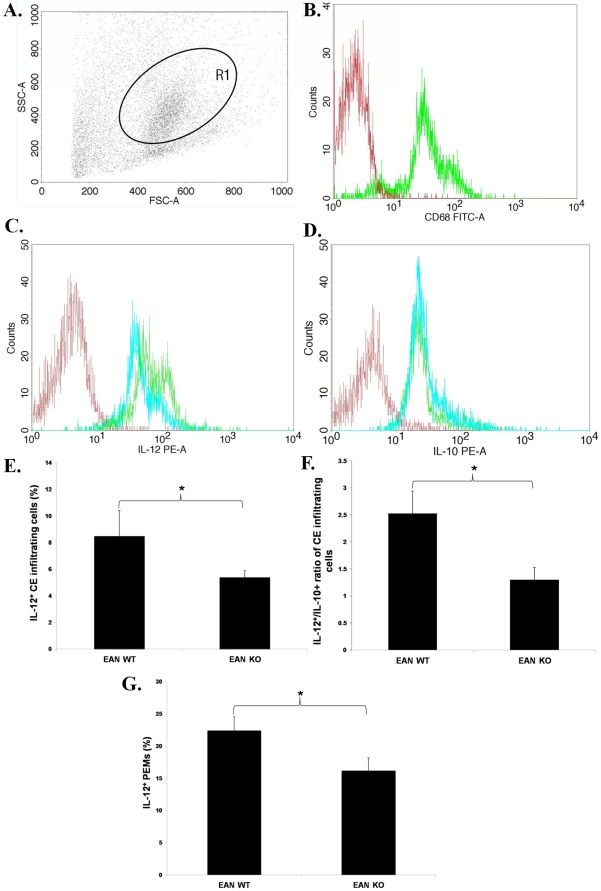
Expression of IL-12 in the CE infiltrating cell and in non-stimulated PEMs of TNF-α KO EAN mice. **A**. EAN mice were sacrificed at nadir of disease (day 28 p.i.) and CE infiltrating cells were analyzed by flow cytometry. Generic features of CE infiltrating cells in EAN, as assessed by light scatter properties (FSC and SSC) in flow cytometry, are shown. **B**. R1 represents macrophage (macrosialin+) subpopulation (The red histogram denotes the staining with FITC-conjugated isotype antibody and the green one denotes the staining with anti-CD68 (macrosialin) antibody (Clone: ED1)). **C**. Representative flow cytometric data show that CE infiltrating cells of TNF-α KO mice expressed lower levels of IL-12 (blue histogram) than those of WT mice (green). The histogram in red denotes the staining with PE-conjugated isotype antibody. **D**. Representative flow cytometric data indicate similar expressions of IL-10 by CE infiltrating cells of TNF-α KO mice (blue histogram) and of WT mice (green). The red histogram denotes the staining with PE-conjugated isotype antibody. **E**. CE infiltrating cells of TNF-α KO mice expressed significantly lower IL-12 levels than those of WT mice. **F**. The ratio of the expressing cells of IL-12 to IL-10 was significantly lower in CE infiltrating cells from TNF-α KO mice than from WT mice. **G**. At the peak of EAN (day 28 p.i.), non-stimulated PEMs were collected by standard lavage of peritoneal cavity and the expression of IL-12 was detected by flow cytometry. TNF-α deficient PEMs expressed significantly lower levels of IL-12 compared with their WT counterparts. Data are presented as mean value ± SD of one representative out of two independent experiments (n = 5 or 6 in each group). * *p*<0.05.

EAN mice were sacrificed at nadir of disease (day 28 p.i.) and CE infiltrating cells were analyzed by flow cytometry (Figure 2A). The infiltrating macrophages (Figure 2B) from TNF-α KO mice exhibited reduced levels of IL-12 (Figure 2C and 2E) and similar levels of IL-10 (Figure 2D), when compared with those from WT mice. Furthermore, the ratio of the percentages of IL-12 expressing cells to that of IL-10 expressing cells was lower in CE infiltrating cells from TNF-α KO mice than from WT mice (Figure 2F).

In addition, at the peak of EAN, non-stimulated PEMs were collected for flow cytometric analysis. TNF-α deficient PEMs expressed reduced levels of IL-12 as compared with their WT counterparts (Figure 2G).

**Figure 3:**
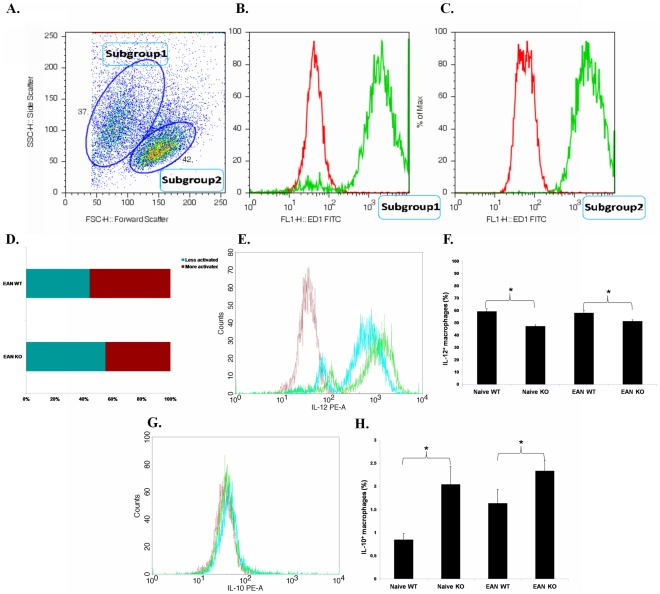
Expression of IL-12 and IL-10 in stimulated macrophages. Thioglycollate elicited macrophages were harvested by standard lavage of peritoneal exudates with 10 ml serum-free culture medium DMEM/F12, cultivated and stimulated with LPS plus IFN-γ for 24 h. **A**. Macrophages were assessed by flow cytometry. In both naïve and EAN mice, two subgroups of PEMs were presented. The right group of cells with larger size (Subgroup 2) expressed higher levels of IL-6 and IL-12 (data not shown), compared with Subgroup 1, indicating a stronger activation of these cells. **B**. and **C**. Cells in both groups expressed macrosialin (The red histograms denote the stainings of controls with FITC-conjugated isotype antibody and the greens denote the stainings with anti-macrosialin antibody (Clone: ED1)). **D**. In EAN, the proportion of more activated PEMs was significantly lower in TNF-α KO mice than in WT mice (*p*<0.05). **E**. Representative flow cytometric data show that after proinflammatory stimulation, macrophages from TNF-α KO mice with EAN (blue histogram) expressed lower levels of IL-12 than from WT mice with EAN (green). The red histogram denotes the staining with PE-conjugated isotype antibody. **F**. After proinflammatory stimulation, the proportion of IL-12^+^ macrophages was significantly lower in TNF-α KO mice than in WT mice (both naive and EAN). **G**. Representative flow cytometric data show that after proinflammatory stimulation, macrophages from TNF-α KO mice with EAN (blue histogram) expressed higher levels of IL-12 than from WT mice with EAN (green). The red histogram denotes the staining with PE-conjugated isotype antibody. **H**. After proinflammatory stimulation, the proportion of IL-10^+^ macrophages was significantly higher in TNF-α KO mice than in WT mice, independent of EAN induction. Data are presented as mean value ± SD of one representative out of three independent experiments (n = 5 or 6 in each group). * *p*<0.05.

Thioglycollate (Fluka, Milwaukee, WI, USA) elicited PEMs were cultured and stimulated with LPS plus IFN-γ for 24 h. Flow cytometry was used to assess cytokine production. Cultured PEMS represented mainly macrophages as tested by labeling with the macrophage marker macrosialin (Figure 3A–3C). Two subgroups of PEMs from EAN mice were present in Figure 3A according to their light scatter properties. The group of cells with larger size expressed higher levels of IL-6 and IL-12 indicating a stronger activation of these cells. The proportion of more activated PEMs was higher in EAN than naïve mice [22]. In EAN, the proportion of more activated PEMs was markedly lower in TNF-α KO mice than in WT mice (Figure 3D). Macrophages from TNF-α KO mice expressed lower levels of IL-12 (Figure 3E and 3F) and higher levels of IL-10 (Figure 3G and 3H) as compared with those from WT mice independent of EAN induction.

**Figure 4:**
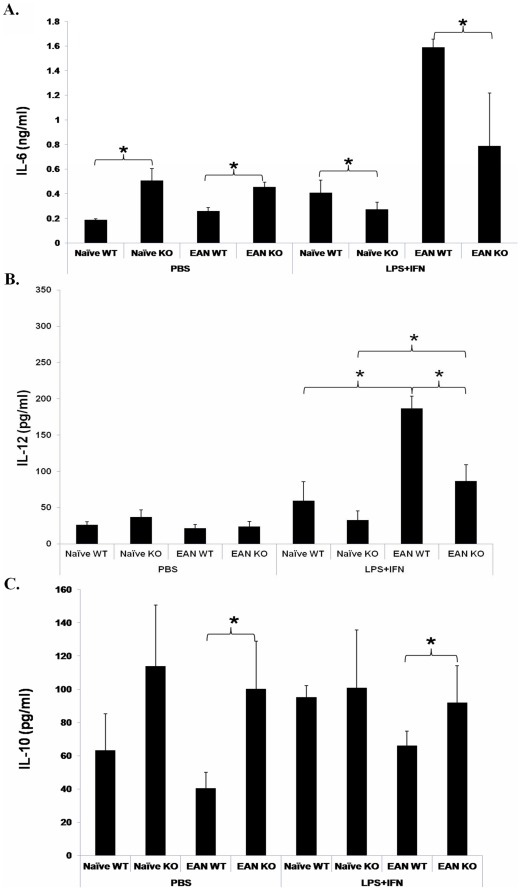
Production of IL-6, IL-10 and IL-12 in the culture supernatants of macrophages. **A**. Macrophages from naïve and EAN mice were cultured with PBS or stimulated with LPS (100 ng/ml) plus IFN-γ (100 IU/ml) for 24 h. Supernatants were collected for ELISA detection. Without stimulation, macrophages from TNF-α KO mice (both naive and EAN mice) produced higher levels of IL-6 than those from WT mice, whereas after stimulation, macrophages from TNF-α KO mice produced lower levels of IL-6 than those from WT mice. **B**. For IL-12, the cytokine level in cultures of stimulated macrophages of TNF-α KO mice with EAN was significantly lower than WT mice with EAN. The level in cultures of stimulated macrophages was also lower in the control group than in the EAN group (for both WT and TNF-α KO mice). **C**. For IL-10, an opposite result to IL-12 was registered, i.e. stimulated and unstimulated macrophages from TNF-α KO mice with EAN produced higher levels of IL-10 than those from WT mice with EAN. As for IL-12, naïve mice showed no significant differences. Data are presented as mean value ± SD of one representative out of three independent experiments (n = 5 or 6 in each group). * *p*<0.01.

Supernatants of PEM cultures were collected for cytokine production assessment. Without stimulation, macrophages from TNF-α KO mice (both naïve and EAN mice) produced higher levels of IL-6 than those from WT mice, whereas after stimulation, macrophages from TNF-α KO mice produced lower levels of IL-6 than those from WT mice (*p*<0.01, Figure 4A). For IL-12 production, the only significant finding was a lower cytokine production by stimulated macrophages from EAN TNF-α KO mice than from EAN WT mice (*p*<0.01, Figure 4B). For IL-10 production, the opposite was the case, i.e. stimulated as well as unstimulated macrophages from EAN TNF-α KO mice with EAN produced higher levels of IL-10 than those from EAN WT mice (*p*<0.01, Figure 4C).

No significant differences were found with regard to the expression of TNFR2, IL-4, MCP-1 and CXCL9, respectively, on or in CE infiltrating cells, as well as with regard to the proportions of αβTCR^+^ and γδTCR^+^ cells relative to total infiltrating cells (data not shown). Likewise, no significant differences were found with regard to the expressions of TNFR2, MCP-1 and CXCL9, respectively, on cultured macrophages (data not shown).

**Figure 5:**
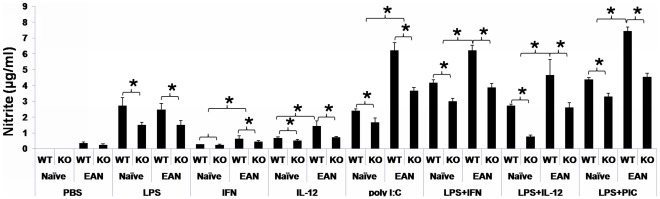
NO production of cultured macrophages. PEMs from naïve and EAN mice were cultured and stimulated with LPS (100 ng/ml), IFN-γ (100 IU/ml), IL-12 (10 ng/ml), poly I:C (50 µg/ml), combinations thereof. In each set of stimulation, NO production was lower in the TNF-α KO group than in the WT group (*p*<0.01 for all comparisons). Data are presented as mean value ± SD of one representative out of three independent experiments (n = 5 or 6 in each group).

Macrophages from naïve and EAN mice were cultured and stimulated with LPS, IFN-γ, IL-12, poly I:C, and combinations thereof. In each set of stimulation, NO production was lower in TNF-α KO group than in the WT group (*p*<0.05 for all comparisons, Figure 5). For the TNF-α stimulated set, NO production was higher in EAN TNF-α deficient macrophages than in EAN WT macrophages (0.92±0.24 μg/ml *versus* 0.44±0.14 μg/ml). Upon TNFR1 blockade, NO was almost undetectable in macrophage cultures, independent of TNF-α gene status and EAN induction.

### TNF-α Deficiency Increases Tregs and Reduces IFN-γ Production

CE infiltrating cells and splenic MNCs were analyzed at nadir of EAN (day 28 p.i.) by flow cytometry. Cell debris was excluded by proper gating. The yield regarding CE infiltrating cells and splenic MNCs was similar between or among groups (data not shown). We routinely detected the proportion of different immune cell subsets in naïve and EAN conditions by using labeled antibodies against CD3, CD4, CD8, CD45, NK1.1, dendritic cell marker, TCR, BCR, macrosialin, etc. No significant difference was seen regarding the proportions of different cell subsets (the CD4^+^ subset, the macrosialin^+^ subset, and the CD8^+^ subset, etc., data not shown).

**Figure 6:**
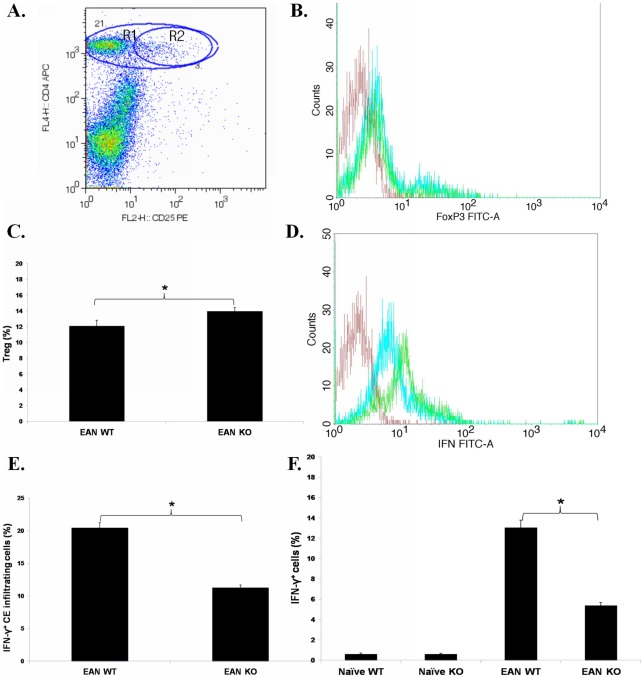
Tregs and IFN-γ production. EAN mice were sacrificed at nadir of EAN (day 28 p.i.) and CE infiltrating cells were analyzed by flow cytometry. **A**. Gating for Treg in CE infiltrating cells was shown. R1 represents CD4^+^ T cells. R2 represents CD4^+^CD25^+^ cells, which were confirmed by FoxP3 staining. **B**. In TNF-α KO mice, the number of FoxP3-expressing cells (blue histogram) is slightly increased compared to WT mice (green). The red histogram denotes the staining with FITC-conjugated isotype antibody. **C**. The percentage of Tregs (CD4^+^CD25^+^FoxP3^+^) relative to CD4^+^ CE infiltrating cells was higher in TNF-α KO than in WT mice with EAN. **D**. Representative flow cytometric data show that infiltrating cells from TNF-α KO mice (blue histogram) expressed lower levels of IFN-γ than from WT mice (green). The red histogram denotes the staining with FITC-conjugated isotype antibody. **E**. The percentage of IFN-γ^+^ cells relative to CD4^+^ CE infiltrating cells was significantly lower in TNF-α KO than in WT mice with EAN. **F**. EAN mice were sacrificed at nadir of EAN (day 28 p.i.) and single cell suspensions of splenic MNCs were analyzed by flow cytometry. The percentage of IFN-γ expressing cells relative to CD4^+^ splenic MNCs was lower in TNF-α KO than in WT mice with EAN. Data are presented as mean value ± SD of one representative out of three independent experiments (n = 5 in each group). * *p*<0.05.

Within the cell gate, CD4^+^TCR^+^ and macrosialin^+^ cells accounted for more than 90% of the total CE infiltrating cells, and the other cells including CD8^+^TCR^+^ cells, dendritic cells, NK cells and B cells accounted for less than 10%. The percentage of Tregs (CD4^+^CD25^+^FoxP3^+^) in CE infiltrating cells was higher in TNF-α KO than in WT mice with EAN (*p*<0.05, Figure 6A–6C). The percentage of IFN-γ expressing cells relative to CD4^+^ CE infiltrating cells was lower in TNF-α KO than in WT mice with EAN (*p*<0.05, Figure 6D and 6E). The same was also true for splenic MNCs in EAN, but not in naïve mice (*p*<0.05, Figure 6F).

### TNF-α Deficiency Decreases Levels of Anti-P0 Peptide 180–199 IgG2b Antibodies

Serum samples of mice at nadir of EAN (day 28 p.i.) were analyzed by ELISA for anti-P0 peptide 180–199 antibodies (IgM, pan-IgG, IgG1, IgG2a and IgG2b). The concentrations of IgG2b (OD values at 450 nm) were significantly lower in TNF-α KO than WT mice with EAN (0.42±0.01 *versus* 0.45±0.01, n = 7, *p*<0.01). No difference was found with regard to serum concentrations of IgM, pan-IgG, IgG1 or IgG2a (data not shown).

### TNF-α Deficiency Upregulates Production of IL-17A

**Figure 7:**
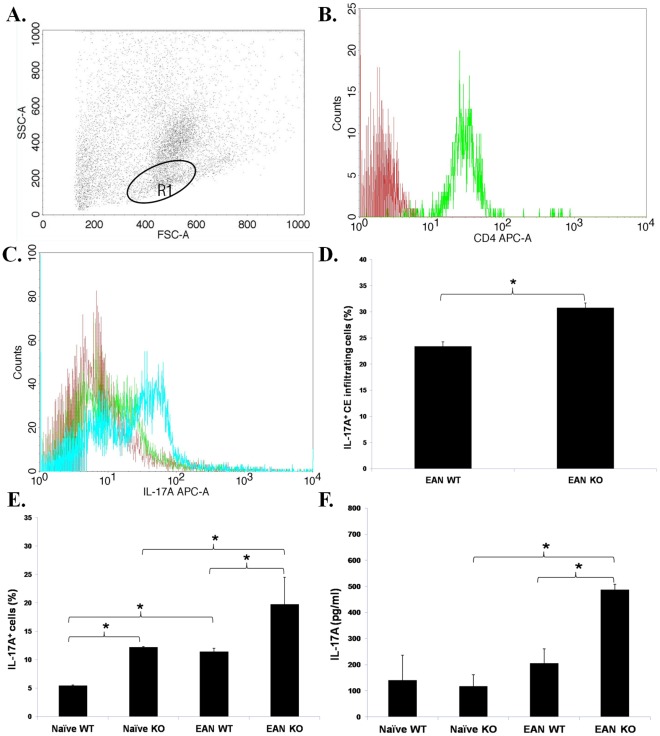
IL-17A production. **A**. EAN mice were sacrificed at nadir of EAN (day 28 p.i.) and CE infiltrating cells were analyzed by flow cytometry. Generic features of the cells as assessed by light scatter properties (FSC and SSC) in flow cytometry, are shown. **B**. R1 represents CD4^+^ cells (>95%). **C**. Representative flow cytometric data show that CD4^+^ CE infiltrating cells from TNF-α KO mice (blue histogram) expressed higher levels of IL-17A than from WT mice (green histogram). The red histogram denotes the staining with APC-conjugated isotype antibody. **D**. The percentage of CD4^+^IL-17A^+^ cells was higher in TNF-α KO than in WT mice with EAN. **E**. Also at nadir of EAN, single cell suspensions of splenic MNCs were analyzed by flow cytometry. The percentage of CD4^+^IL-17A^+^ cells in spleen was elevated in EAN compared to naïve mice. Furthermore, it was significantly higher in TNF-α KO mice than in WT mice. **F**. Serum samples at nadir of EAN (day 28 p.i.) were obtained and assessed. The concentrations of IL-17A were higher in EAN TNF-α KO mice than in naïve TNF-α KO mice and higher in TNF-α KO mice than in WT mice with EAN. Data are presented as mean value ± SD of one representative out of three independent experiments (n = 6 in each group). * *p*<0.05.

At nadir of EAN (day 28 p.i.), CD4^+^ CE infiltrating cells (Figure 7A and 7B) contained a higher percentage of IL-17A^+^ cells in TNF-α KO than in WT mice (Figure 7C and 7D). At the same time-point single cell suspensions of splenic MNCs contained a higher percentage of CD4^+^IL-17A^+^ cells (Figure 7E), when the TNF-α gene was knocked out. The same was also true for naïve mice, but at a lower level. Similarly, the concentrations of IL-17A in sera were higher in TNF-α KO mice than in WT mice, when EAN mice are regarded. This is in agreement with the finding of a higher serum level of IL-17A in EAN TNF-α KO compared with naïve TNF-α KO mice (Figure 7F).

### The Proinflammatory Role of TNF-α in EAN is Mediated by TNFR1

**Figure 8:**
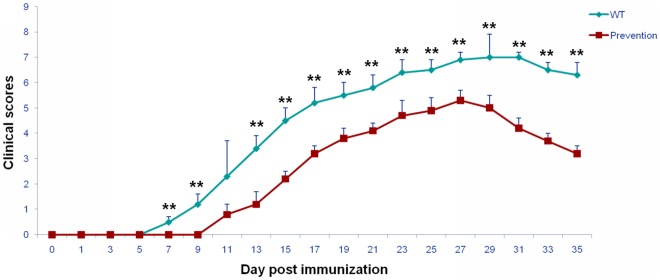
Anti-TNFR1 monoclonal antibodies attenuated the clinical course of EAN in WT mice. EAN was induced in WT mice by immunization with P0 peptide 180–199 in combination with FCA. Anti-TNFR1 monoclonal antibodies were administered intravenously at the dose of 200 µg/kg body weight on days −1, 3, and 7 p.i., respectively. Sex- and age-matched mice from the same strain received 0.9% saline as controls. Using a blinded protocol, two different examiners evaluated clinical severity of EAN mice immediately before immunization (day 0 p.i.) and thereafter every two days until day 35 p.i. From day 7 p.i., the clinical severity was significantly milder in the anti-TNFR1-treated group than in the control group, except on day 11 p.i., when the difference did not reach statistical significance. Data are presented as mean value ± SD of one representative out of two independent experiments (n = 5 in each group). ** *p*<0.01.

To further elucidate which receptor is responsible for the detrimental role of TNF-α in the pathogenesis of EAN, we blocked the functions of TNFR1 with neutralizing antibodies against TNFR1 in WT mice. The onset of EAN in the anti-TNFR1 treated group was later than in saline-treated controls (9–13 days *versus* 7–9 days p.i., Figure 8). EAN reached the peak of the disease on day 30 p.i. in the intervention group and on day 27 p.i. in the control group, respectively. From day 7 p.i., the clinical severity was significantly milder in the anti-TNFR1-treated group than in the control group, except on day 11 p.i., when the difference did not reach statistical significance (Mann-Whitney u-test, Figure 8).

To corroborate the detrimental role of TNFR1 in EAN, we also blocked the functions of TNFR1 with neutralizing antibodies against TNFR1 in both TNF-α KO and WT control mice with EAN. Neither was any significant difference found between groups as to the onset, the peak or the clinical severity before day 35 p.i., nor as to the percentage of Tregs, IFN-γ expressing CD4^+^ cells or the ratio of the percentages of IL-12 expressing cells to that of IL-10 expressing cells in CE infiltrating cells (data not shown).

## Discussion

By inducing EAN in TNF-α KO mice, we found that TNF-α deficiency attenuated the clinical severity of EAN. In accordance with prior studies, our data support a predominantly detrimental role of TNF-α in the pathogenesis of EAN. As an important Th1 cytokine, TNF-α contributes to a Th1-polarized immune response in EAN. TNF-α deficiency may lead to an amelioration of the Th1 immune response, thereby attenuating the clinical severity of EAN. This is evidenced by a downregulated production of IFN-γ and a reduced serum level of anti-P0 peptide 180–199 IgG2b antibodies in EAN TNF-α KO mice.

TNF-α signals through TNFR1 and TNFR2, mainly TNFR1, to elicit partly opposite reactions in multiple cell types [23]. The dual effect of TNF-α segregates at the level of the two different receptors. We found that TNF-α KO mice were not completely resistant to EAN induction. The reason could be that TNF-α is not a uniquely necessary inflammatory molecule for the induction of EAN.

Previously we found conflicting results with regard to the role of TNFR1 in EAN by using TNFR1 knockout mice [24], [25]. The discrepancy has been discussed in the latter publication [25]. Moreover, TNFR2 may compensate for the loss of TNFR1 roles in TNFR1 KO mice, since TNFR1 and TNFR2 are known to trigger overlapping intracellular signaling events (e.g. nuclear factor kappa B) [26]. Regretfully, the functions of macrophages were not investigated in those two studies. However, in experimental autoimmune encephalomyelitis (EAE), an analogous experimental disorder of EAN in the central nervous system, TNFR1 KO mice were totally resistant to EAE, exhibiting reduced antigen-specific proliferative responses and Th1 cytokine production, whereas TNFR2 KO mice exhibited exacerbated EAE, enhanced Th1 cytokine production, and enhanced macrophage and T cell infiltration [27]. To further elucidate which receptor is responsible for the detrimental effect of TNF-α, we blocked the functions of TNFR1 in WT mice with EAN by using anti-TNFR1 monoclonal antibodies. The clinical severity of EAN was markedly mitigated after administration of this TNFR1 antagonist. However, this might also be due to the beneficial role of TNF-α acting via TNFR2 [28], [29]. Evidence showed that the antiinflammatory effects of TNF-α are mediated via TNFR2 in macrophages [30]. We then blocked the functions of TNFR1 in both TNF-α KO and WT mice with EAN and corroborated the detrimental role of TNFR1 in EAN.

Two main effector cells, T cells and macrophages, play critical roles in the pathogenesis of EAN. However, TNF-α deficiency did not change lymphocyte proliferation in response to antigenic or mitogenic stimulant. Hence, we hypothesized that TNF-α deficiency ameliorates the clinical severity of EAN via modulating macrophage functions. Thus, we further focused our interest on macrophages.

Activated macrophages can be divided into two distinct subsets: classically activated macrophages (M1) and alternatively activated macrophages (M2). Th1 cytokines such as IFN-γ and IL-1β, and Toll-like receptor agonists such as LPS and poly I:C induce the M1 phenotype, which is characterized by enhanced production of proinflammatory cytokines, e.g. IL-12, expression of MHC II molecules, and generation of free radicals including NO [31]. M1 macrophages are considered to mediate host defence to infections, but also to cause autoimmune tissue damage. M2 macrophages are induced by Th2 cytokines and express high levels of antiinflammatory molecules, e.g. IL-10, which underlines their role in antiinflammation and tissue repair. A switch of macrophage phenotype from M1 to M2 during EAN could favor the outcome of EAN [32]. Compound A, a plant origin ligand of glucocorticoid receptors attenuated EAN by increasing M2 macrophages [32]. We found a reduced ratio of IL-12/IL-10 in CE infiltrating cells and a lower level of IL-12 in unstimulated PEMs from TNF-α KO mice with EAN, indicating the proinflammatory M1 phenotype of macrophages in EAN was down-regulated due to the TNF-α deficiency.

To gain a deeper insight into the altered functions of macrophages in TNF-α KO mice, we purified and cultivated macrophages *in vitro* and manipulated the culture milieu by adding various inflammatory stimuli. Before cultivation we found two subgroups of macrophages with different light scatter properties, i.e. larger, more activated *versus* smaller, less activated macrophages. The proportion of more activated macrophages (higher levels of IL-6 and IL-12 expression) was lower in naïve than EAN mice and lower in TNF-α KO than WT mice with EAN. After *in vitro* stimulation with proinflammatory agents, macrophages from TNF-α KO mice with EAN produced reduced levels of IL-12 and IL-6 and increased levels of IL-10 compared with those from WT mice with EAN. Moreover, a reduced production of NO was detected in culture supernatants of macrophages from TNF-α KO mice after proinflammatory stimulation. Previously, we reported that 1400 W, a specific inducible nitric oxide synthase (iNOS) inhibitor could remarkably suppress the clinical course of EAN [22]. The reduced production of NO in TNF-α KO mice may explain the attenuated clinical severity of EAN. Taken together, these findings indicate that TNF-α deficiency results in a shift to the M2 phenotype or in a down-redulation of the M1 phenotype after proinflammatory stimulation.

Th17 cells, which are characterized by production of IL-17, have been shown to be independently capable of driving EAE [33] **.** Interestingly, we found an upregulated production of IL-17A in EAN *versus* naïve mice irrespective of the TNF-α gene status and in TNF-α KO mice *verus* WT mice (both naïve and EAN). Since Th1 cytokines antagonize the functions of Th17 cells [34], the mitigated Th1 responses due to TNF-α deficiency might lead to the enhanced Th17 response in TNF-α KO mice. Moreover, the increased levels of IL-17A under EAN conditions compared to naïve conditions, are strongly suggestive of a role of IL-17A in the pathogenesis of EAN. In our another study, we found that IFN-γ deficiency exacerbated EAN via upregulating Th17 cells despite a mitigated Th1 immune response [35]. The elevated levels of IL-17A production may partially explain the incompletely inhibited clinical course of EAN by TNF-α deficiency. Similar findings have been reported in the treatment of the animal model of rheumatoid arthritis with TNF-α and TNFR1 neutralizing antibodies [36]. Further studies are warranted to explore the clinical implications of the increased levels of IL-17A in EAN under TNF-α deficient conditions.

In summary, TNF-α deficiency and TNFR1 blockade remarkably attenuated the clinical severity of EAN. Our findings indicate that TNF-α exacerbates EAN by inducing the M1 phenotype of macrophages. The proinflammatory role of TNF-α in EAN is probably mediated mainly by TNFR1.
